# Sustained drug-delivery system: a promising therapy for denture stomatitis?

**DOI:** 10.1590/1678-77572016ed003

**Published:** 2016

**Authors:** Karin Hermana Neppelenbroek

Dear Readers,

Denture-induced stomatitis is the most common type of oral candidosis and the most frequent mucosal alteration associated with complete or removable partial dentures in the elderly. Despite being an infection of multifactorial etiology, this condition has as main etiological factor the colonization of denture-bearing mucosa and acrylic bases by species of *Candida* spp., especially *Candida albicans,* found in 50 to 98% of all cases^25^.

Different treatments are indicated for denture stomatitis, including topical antifungal and systemic therapy, care with oral hygiene, denture cleaning and disinfection procedures, replacement of old dentures, elimination of anatomic irregularities, re-establishment of atraumatic occlusion, removing the denture at night and nutritional restitution^12^. Although systemic antifungal therapy is suggested for immunosuppressed patients, these drugs may present potential hepatotoxic and nephrotoxic effects and interaction with other drugs, thus increasing the adverse systemic effects^6^. Topical antifungal agents as nystatin and miconazole are largely used for the treatment of denture stomatitis^12,15^. These antifungal drugs are effective in relieving the clinical signs and symptoms of denture stomatitis associated with *Candida* spp.; however, they cannot reach a therapeutic antifungal concentration on the inner denture surfaces^15^. Consequently, re-infection of the treated oral mucosa may occur after conventional therapy with topical and systemic antifungal drugs. The high rates of clinical relapse and recurrence in up to two weeks post-treatment make the treatment of denture stomatitis challenging^15,18^.

Factors other than inability to maintain therapeutic concentrations of antifungal drugs on the surface of dentures are associated with failure of conventional antifungal therapy including the following: 1) reduction of antifungal action by salivary flow and swallowing, and tongue movements; 2) lack of patient compliance to antifungal therapy due to costs required for the medications, unpleasant taste of topical agents, continuous utilization of dentures and strict drug regimen; 3) persistent contact between injured mucosa and contaminated internal denture surfaces, which favors re-infection of the mucosa and causes trauma to supporting tissues, extending the clinical course of the pathology^20,21^.

An effective treatment of denture stomatitis requires a therapy based on the sustained release of antifungal drugs that may reach adequate therapeutic concentration to significantly reduce the *Candida* from both the supporting tissues and infected denture surfaces. In this context, incorporation of antifungal/antimicrobial agents into denture base materials to be progressively released to the oral cavity has been suggested to prevent biofilm accumulation, inhibit *C. albicans* colonization, and contribute to the treatment of denture stomatitis^5,20,21^. This protocol requires only the use of dentures by patients, thus reducing the need for patient compliance to antifungal drug regimens^21^. Furthermore, the incorporation of drugs into denture liners breaks the contact between the denture biofilm and infected tissues, thus avoiding a cycle of re-infection via prostheses^14^. In this regard, the use soft lining materials is highly recommended as it results in the recovery of injured tissues and patient comfort^14^. However, soft lining materials, mainly short-term ones as tissue conditioners and temporary resilient liners are easily degradable and susceptible to microbial colonization^17^. Therefore, the modification of these temporary materials by antimicrobial agents also has the advantage of increasing their clinical longevity. As life cycle of short-term soft liners is approximately 14 days, the treatment period of denture-induced stomatitis using these materials modified by drugs is similar to the period required when conventional topical antifungal agents are used^20,21^. As a result, denture stomatitis can be treated before replacing temporary soft liners with long-term liners or fabricating new dentures, in a relatively short period.

Although incorporation of antifungal/antimicrobial agents at commercially available concentrations to polymeric/plastic materials can effectively inhibit the growth of *C. albicans* ^20,21^, it may affect their morphological structure^24^ and properties such as tensile strength^1,22^, water absorption^7^, modulus of elasticity and weight^1^, hardness^1,20,23^, roughness^23^, and peel bond strength to denture base resin^2^. In an order to provide lower concentrations of drugs to these materials without severely compromising their properties, Bueno, et al.^5^ determined minimum inhibitory concentrations (MICs) of *C. albicans* biofilm for antifungal/antimicrobial agents added to a temporary resilient liner and a tissue conditioner. The five drugs (nystatin, miconazole, ketoconazole, itraconazole, and chlorhexidine diacetate) incorporated at MICs into both soft materials were effective in inhibiting the growth of *C. albicans* for up to 14 days.

Considering the promising results of Bueno, et al.^5^, some studies were performed to evaluate the effects of drug addition at MICs on important properties of these materials. With the exception of itraconazole, the MICs of drugs incorporated into temporary soft lining materials resulted in minimal changes in their peel bond strength to a denture base resin within 14 days^19^. After 14 days, the MICs of nystatin and ketoconazole in both materials and chlorhexidine in temporary resilient liner did not significantly influence their water sorption^10^. The solubility of both resilient materials was not modified by nystatin at MIC within 14 days^10^. Also, in this issue, Lima, et al.^11^ observed that the addition of drugs at MICs resulted in no harmful effects for the porosity of both soft lining materials in different periods of water immersion, except for chlorhexidine and nystatin in the tissue conditioner and chlorhexidine in the temporary resilient liner at 14 days. Despite these favorable outcomes, before the incorporation of drugs at MICs may be indicated as an alternative therapy for denture stomatitis, it is necessary to evaluate the biocompatibility of this protocol with the oral tissues. During their life cycle, polymeric/plastic materials release soluble substances in the oral environment, which may be potentially toxic, such as methyl methacrylate and dibutyl phthalate. When released in saliva, these components may even act at sites distant from the area contacting the material^9,16^. These possible cytotoxic effects have been assessed *in vitro* by using mouse fibroblast culture^9,16^, which is clinically restricted, since the effects of tests performed directly on cells are more marked than the oral conditions *in vivo.* In addition, the limited literature available on the in vivo biocompatibility of denture base liners with oral tissues in animal models showed an increased thickness of the stratum corneum layer for rats receiving acrylic intraoral device relined with non-modified temporary resilient material^3,4^. Nevertheless, there is lack of information on the biocompatibility of drug-modified denture liners with oral tissues in animal models.

From the *in vitro* studies evaluated, it is possible to conclude that the modification of temporary resilient materials by antifungal/antimicrobial agents, especially in lower concentrations, may represent a viable protocol for *in vivo* treatment of denture stomatitis during a period similar to the conventional therapy with topical antifungals (14 days). However, it is important to emphasize that when in the mouth, denture resilient liners may be subjected to additional thermal stress, pH range, and occlusal load, which could lead to other pattern of properties of these products. This might explain the evidence that the magnitude and speed of all changes in material properties after immersion in distilled water (as done in all *in vitro* studies on the properties of modified soft liners) are different from those observed in clinical condition^13^. Nevertheless, no evidence was found to confirm how each of these factors affect the evaluated properties. Moreover, the loss of leachable components is faster when the soft liner is in the mouth due to the oral environment, food, and cleaning methods adopted by the patient^13^.

Considering the aspects described previously, before clinical indication of this protocol for denture stomatitis treatment, future *in vitro* studies are necessary to evaluate other relevant properties of modified soft liners such as daily drug release and drug pattern of incorporation. Furthermore, the findings of animal model studies on temporary resilient liners should not be considered enough for clinical indication of a material or treatment, since important factors as removal and hygiene of the appliances and deformation due to occlusal load in normal feeding conditions were not analyzed. Thus, after observations of *in vitro* studies on other properties of modified materials as well *in vivo* studies on animal models testing this protocol, clinical trials in humans are necessary to allow the safe indication of drug incorporation in temporary resilient liners as optimal antifungal delivery for denture stomatitis treatment.
